# Vasospasm following low-velocity penetrating pediatric intracranial trauma

**DOI:** 10.1186/s13256-022-03254-5

**Published:** 2022-02-06

**Authors:** Alysa Almojuela, Zul Kaderali, James McEachern, Colin Kazina, Demitre Serletis

**Affiliations:** 1 Section of Neurosurgery, Department of Surgery, University of Manitoba, Winnipeg, MB Canada; 2 Division of Neurosurgery, Royal Columbian Hospital, New Westminster, BC Canada; 3 Department of Radiology, University of Manitoba, Winnipeg, MB Canada; 4 Department of Neurosurgery, Cleveland Clinic Foundation, Cleveland, OH USA; 5 Charles Shor Epilepsy Center, Neurological Institute, Cleveland Clinic, Cleveland, OH USA

**Keywords:** BB gun, Pediatric, Penetrating brain injury, Cerebral vasospasm, Case report

## Abstract

**Background:**

BB guns or non-powder guns created in the modern era are able to reach exceedingly fast velocities as a result of advances in compressed-gas technology. While missile penetrating trauma has been well documented in neurosurgical literature, penetrating intracranial injury secondary to non-powder guns, along with their associated complications and treatments, is not well described, and even less so in the pediatric population.

**Case presentation:**

Here, we describe an unusual case of a 6-year-old Indigenous child who was accidentally shot with a BB gun to the head. He subsequently developed delayed acute right-sided weakness due to symptomatic vasospasm. His symptoms resolved following hypertensive therapy, balloon angioplasty intervention, and intra-arterial milrinone therapy.

**Conclusions:**

This case highlights the unique complication of delayed symptomatic vasospasm in a child following a non-missile intracranial penetrating injury. Intracranial vasospasm needs to be considered in the presence of delayed neurological deficit given its potential reversibility. This case also emphasizes the importance of gun safety and education when handling and storing these potential weapons around children.

**Supplementary Information:**

The online version contains supplementary material available at https://doi.org/10.1186/s13256-022-03254-5.

## Background

Penetrating intracranial injuries pose unique challenges to medical and neurosurgical management, particularly in the pediatric patient cohort and in low-velocity type injuries, such as those inflicted by BB guns (air guns used to fire metallic ball projectiles or pellets). Between 2003 and 2013 in the USA, 200,645 nonfatal pellet gun injuries were reported, with nearly two-thirds (127,742) occurring in children [1]. Further, hospitalizations due to injuries inflicted by non-powder guns cost more than 10 million US dollars annually; of hospitalized patients, half require a major surgical procedure [2]. Despite the potential morbidity associated with non-powder guns, approximately 3.2 million BB and/or pellet guns continue to be sold in the USA every year [3], and while variable local laws exist in some states, there are no federal laws to regulate their sales or use [2].

Whereas high-velocity injuries are often fatal, low-velocity injuries such as those caused by non-powder guns are less likely to be lethal and are associated with complications such as infection, pseudoaneurysm formation, and, rarely, symptomatic vasospasm. This cohort of patients therefore requires special consideration for follow-up and management by health professionals, including neurosurgeons. Here, we present an unusual case of a child who sustained a low-velocity penetrating intracranial injury from a BB gun, which caused symptomatic intracranial vasospasm, and was successfully treated with hypertensive therapy, balloon angioplasty, and intra-arterial milrinone therapy.

## Methods

Formal approval to publish this case report was obtained from the authors’ institutional research ethics board. Consent was also obtained from the patient’s mother to publish the study as a case report. The intent of the study and possible risks of publication related to patient confidentiality were discussed.

A literature review was conducted by searching PubMed for studies related to pediatric low-velocity penetrating intracranial injury, BB guns, pellet guns, and intracranial vasospasm. The retrieved studies, which included reviews, case series/reports, and retrospective studies, were reviewed by the primary author.

## Case presentation

### Patient presentation

**Fig. 1:**
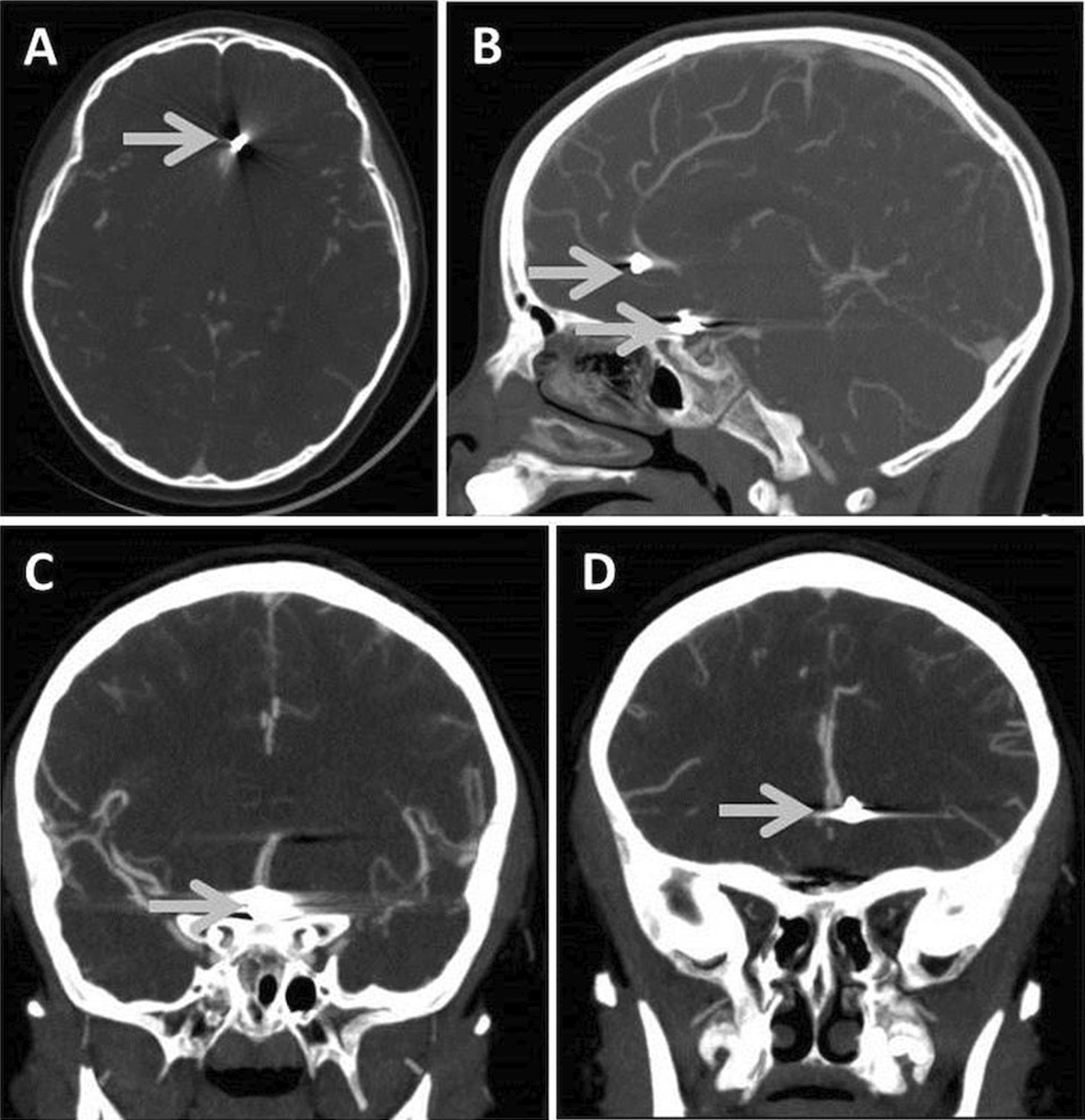
Axial (**A**), sagittal (**B**), and coronal (**C**, **D**) views of CTA study, confirming the location of two intracranial, metallic shrapnel fragments from a BB pellet gun injury (shrapnel demarcated by gray arrows)

A 6-year-old, previously healthy Indigenous male was accidentally shot in the left supraorbital area with a BB gun while playing at home. He was emergently flown to a tertiary trauma center for further management. On examination, his Glasgow Coma Scale (GCS) was 13 (E3V4M6). He displayed no focal neurological deficits. A small entry wound was visible just superior to his right eye, with no exit wound identified. Urgent computed tomography (CT) imaging (including CT angiography, or CTA) revealed two intracranial foreign bodies (that is, shrapnel), with surrounding subarachnoid hemorrhage in the Sylvian fissures, suprasellar cistern, ambient cisterns, and frontal lobes (Fig. 1A–D). Ventricular caliber was normal, and there was no evidence of parenchymal injury or ischemic stroke. CTA of the circle of Willis was limited due to metallic streak artifacts, but confirmed proximity of one piece of shrapnel 2 mm lateral to the left A2 segment in the frontal lobe. The second foreign body localized to the region of the anterior communicating artery. The distal vasculature appeared intact, with no signs of vasospasm. An early cerebral angiogram was performed, confirming these findings.

Given his neurological and hemodynamic stability, the patient was admitted for close clinical observation. He was started on phenytoin for seizure prophylaxis and antibiotic therapy (that is, empiric ceftriaxone, vancomycin, and metronidazole), given the nature of the penetrating traumatic injury. Seven days following admission to hospital, the patient acutely developed right-sided weakness affecting his arm (motor grade 4/5) and leg (motor grade 4+/5). He was otherwise hemodynamically stable, with minimal headache and normal level of consciousness. Urgent CTA imaging revealed narrowing of the left middle and anterior cerebral arteries, along with the supraclinoid portion of the left internal cerebral artery. The patient was thus transferred to the intensive care unit to initiate hypertensive therapy for vasospasm. Maintenance intravenous fluids were started, using fluid boluses and norepinephrine to maintain a mean arterial pressure (MAP) of greater than 90 mmHg, which improved his weakness.

**Fig. 2:**
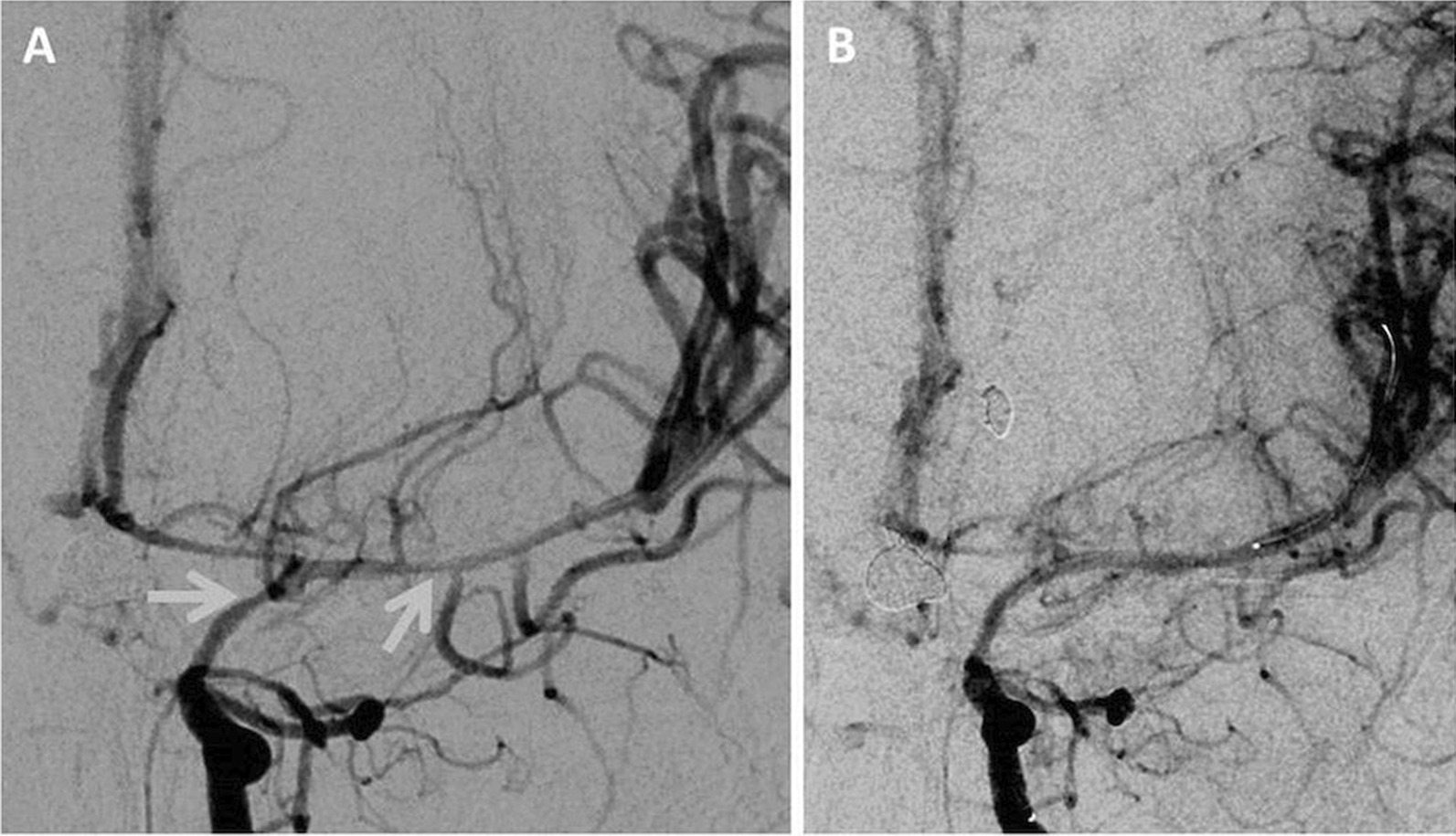
**A** Anteroposterior view of a cerebral angiogram study showing radiographic evidence for vasospasm, with narrowing identified at the supraclinoid portion of the left internal cerebral artery and along the left middle cerebral artery (shown by gray arrows). **B** Radiographic improvement following injection of intra-arterial milrinone and balloon angioplasty

The following day, though his weakness was improved, the patient became disoriented and developed expressive speech dysarthria. A cerebral angiogram was performed showing persistent vasospasm in the affected vessels, and balloon angioplasty of the left internal carotid, left middle cerebral, left anterior cerebral, and right anterior cerebral arteries was subsequently performed, as shown in Fig. 2A, B. Five milligrams of intra-arterial milrinone were delivered, and the patient was started on nimodipine at a dose of 15 mg every 6 hours (roughly 1.5 mg per kg per day). His MAP was maintained at greater than 90 mmHg for 3 days, then greater than 85 mmHg for 4 days, totaling 7 days of MAP-directed therapy. The patient’s symptoms resolved, and repeat CTA imaging revealed radiographic resolution of the previously identified vasospasm in the affected vessels. The patient remained stable and was discharged from hospital 6 weeks post-admission.

## Discussion and conclusions

Many of today’s modern compressed-gas weapons generate enough muzzle velocity to easily penetrate the adult skull. While a common pellet weighing approximately 0.5 g requires a minimum velocity of only 825 ft/second to penetrate the adult skull, some commercially available non-powder guns have muzzle velocities upwards of 1250 ft/second [3]. Moreover, there is a greater propensity for children to sustain severe injuries from these weapons secondary to their thinner skulls and soft tissues [3]. This case highlights the unique vascular complication of vasospasm following an unusual low-velocity penetrating intracranial injury in a child.

In general, the most common vascular complication reported in low-velocity penetrating intracranial injuries is traumatic pseudoaneurysm formation, typically developing within the first 2–3 weeks of injury, but also known to occur in a delayed fashion [3–5]. Whereas pseudoaneurysm formation is a well-known sequela to this type of injury [4], vasospasm has been less studied in this context, and even less so in the pediatric population. Studies suggest that the onset of vasospasm typically begins between 2 and 5 days after injury, as in aneurysmal subarachnoid hemorrhage [6–8]. In the setting of traumatic subarachnoid hemorrhage, the underlying mechanism for the development of vasospasm largely relies on inflammatory pathways. As blood products are broken down by phagocytosis, free radical oxygen species are generated. These species then scavenge electron donors from the lipid bilayers of vascular endothelial and smooth muscle cell membranes, leading to dysfunction of vascular walls and upregulation of vasoconstrictors such as prostaglandins, serotonins, and thromboxin [7, 9]. These physiological changes collectively result in a net vasoconstrictive effect (that is, vasospasm) in the affected vessels, within the vicinity (and beyond) of the subarachnoid hemorrhage. The development of vasospasm in the case presented here may have been attributable to a combination of subarachnoid hemorrhage, direct vascular irritation or damage, and local inflammation due to the retained pellet fragments.

Importantly, the incidence and prevalence of vasospasm following traumatic brain injury has not been thoroughly studied, with only small cohorts reported in the literature. Kordestani *et al.* identified a 42.4% incidence of vasospasm using transcranial Doppler following cranial gunshot wounds in patients aged 15–50 years [6], and showed a trend towards favorable neurological outcome in patients without vasospasm. A favorable Glasgow Outcome Score (that is, good recovery or moderate disability) was observed in 47.4% of patients without evidence of vasospasm at least 3 months following injury, compared with a lower incidence (35.7%) in those with vasospasm. Although not statistically significant (*p* = 0.12), this suggests that post-traumatic vasospasm may play a substantial role in the worsened outcomes reported for cases of low-velocity penetrating intracranial injury [6].

Within the pediatric literature, a prospective study of 69 children with traumatic brain injury [7] showed a prevalence of middle cerebral artery vasospasm (as diagnosed via Doppler ultrasound) of 8.5% in moderate traumatic brain injury patients (defined by GCS 9–12), and 33.5% in patients with severe traumatic brain injury (GCS ≤ 8). Similarly, the prevalence of basilar artery vasospasm in moderate brain injury patients was 3%, and much higher (21%) in severe brain injury patients. Again, there was a trend towards improved neurological outcome in patients without vasospasm. In moderate traumatic brain injury, good neurological outcome (as defined by a pediatric-based Glasgow Outcome Score-Extended ≥ 4 at 1 month post-injury) was seen in 76% in patients without evidence for vasospasm, as compared with 40% in those with diagnosed vasospasm. In severe traumatic brain injury patients, good outcomes were observed in 29% of patients without vasospasm, as compared with only 15% of patients with diagnosed vasospasm [7].

Despite a relatively high prevalence of vasospasm diagnosed via diagnostic imaging, symptomatic vasospasm is thought to be rare. A retrospective study of 37 children with subarachnoid hemorrhage [10] demonstrated the prevalence of vasospasm to be 46% (as determined by angiography); despite this, only three children (8%) manifested with clinically symptomatic vasospasm, all of whom had poor collateral vessels on vascular imaging. Thus, excellent collateralization of cerebral vasculature may be protective against vasospasm in children. Other factors that may explain some resilience in the pediatric cohort to develop clinically significant vasospasm may relate to clinical hemodynamic fluctuations, as well as the resilience of molecular pathways regulating the synthesis of vasoconstrictors and vasodilators such as nitric oxide [10].

Treatment of traumatic vasospasm is complex, as the standard treatment of hypertension, hypervolemia, and hemodilution often used in aneurysmal vasospasm can be detrimental in trauma patients with coexistent cerebral edema, and should be pursued cautiously [11]. Nimodipine, a dihydropyridine calcium channel blocker, is a standard part of therapy following aneurysmal subarachnoid hemorrhage in adults to prevent morbidity associated with delayed cerebral ischemia [12]. In children, robust studies evaluating the efficacy of nimodipine are lacking; however, it may be considered in the management of vasospasm [13]. Angioplasty and intra-arterial vasodilators have also been well described as effective treatment options in aneurysmal causes of vasospasm [14] but have not been thoroughly evaluated with respect to post-traumatic etiologies. These treatment modalities were extrapolated from the adult aneurysmal vasospasm literature and employed successfully in our case; however, their routine use in post-traumatic pediatric cases has yet to be rigorously studied. The utility of removing any foreign bodies to prevent vasospasm has also not been thoroughly studied and was avoided in this case owing to the patient’s hemodynamic and neurologic stability.

Aside from vasospasm, important nonvascular complications following low-velocity penetrating intracranial injuries include infection [5, 15], with theoretically higher rates in low-velocity injuries where the penetrating pellet is considered unsterile. Moreover, post-traumatic seizures, known to arise in 30–50% of patients [15], are also common. Additional long-term consequences to consider include intracranial migration of BB pellets into other critical neurovascular structures, and lifelong contraindication to magnetic resonance (MR) imaging [3].

Strengths of this case report include the uniqueness of the case and the use of successful vasospasm treatment that resolved the patient’s symptoms. This case report also has educational value in that it is hypothesis generating for the ideal treatment of traumatic intracranial vasospasm in pediatrics. Limitations include the single patient case and, thus, lack of generalizability. Given our single case, further studies are required to demonstrate the effectiveness of treatment (Additional file 1).

Overall, low-velocity penetrating intracranial injuries in the pediatric population are a source of significant morbidity. Symptomatic vasospasm in this context has not been well described, and its optimal treatment is unclear. Here, we describe a rare pediatric case exemplifying the unique complication of symptomatic vasospasm in the context of a penetrating injury by a low-velocity BB gun. Based on the findings presented here, vasospasm should be considered in the presence of a delayed onset of neurological deficits, and early management should be initiated given its potential reversibility.

## Supplementary Information

**Additional file 1. **An additional file contains the completed CARE Checklist.

## Data Availability

Not applicable.

## Abbreviations

MR: Magnetic resonance
GCS: Glasgow Coma Scale
CT: Computed tomography
MAP: Mean arterial pressure
mmHg: Millimeters of mercury
ft/sec: Feet per second
